# OligoPrime: An Information System for Oligonucleotide Management

**DOI:** 10.1177/11795972211041983

**Published:** 2021-09-08

**Authors:** Šimen Ravnik, Ines Žabkar, Uršula Prosenc Zmrzljak, Ivana Jovčevska, Neja Šamec, Miha Moškon, Alja Videtič Paska

**Affiliations:** 1 Faculty of Computer and Information Science, University of Ljubljana, Ljubljana, Slovenia; 2 Faculty of Medicine, University of Ljubljana, Ljubljana, Slovenia; 3 Labena D.O.O., Ljubljana, Slovenia; 4 Institute of Biochemistry and Molecular Genetics, Faculty of Medicine, University of Ljubljana, Ljubljana, Slovenia

**Keywords:** Oligonucleotide, primer, information system, management, computational support, web application

## Abstract

With the increasing number of molecular biology techniques, large numbers of oligonucleotides are frequently involved in individual research projects. Thus, a dedicated electronic oligonucleotide management system is expected to provide several benefits such as increased oligonucleotide traceability, facilitated sharing of oligonucleotides between laboratories, and simplified (bulk) ordering of oligonucleotides. Herein, we describe OligoPrime, an information system for oligonucleotide management, which presents a computational support for all steps in an oligonucleotide lifecycle, namely, from its ordering and storage to its application, and disposal. OligoPrime is easy to use since it is accessible *via* a web browser and does not require any installation from the end user’s perspective. It allows filtering and search of oligonucleotides by various parameters, which include the exact location of an oligonucleotide, its sequence, and availability. The oligonucleotide database behind the system is shared among the researchers working in the same laboratory or research group. Users might have different roles which define the access permissions and range from students to researchers and primary investigators. Furthermore, OligoPrime is easy to manage and install and is based on open-source software solutions. Its code is freely available at https://github.com/OligoPrime. Moreover, an implementation of OligoPrime, which can be used for testing is available at http://oligoprime.xyz/. To our knowledge, OligoPrime is the only software solution dedicated specifically to oligonucleotide management. We strongly believe that it has a large potential to enhance the transparency of use and to simplify the management of oligonucleotides in academic laboratories and research groups.

## Introduction

One of the most common and essential techniques in biology and medicine is the polymerase chain reaction (PCR). It is routinely used in various approaches, from classical PCR to more sophisticated quantitative real-time PCR (RT-qPCR) all the way to the preparation of next-generation sequencing (NGS) libraries. Since the cost of PCR is affordable to all kinds of laboratory settings, the amount and diversity of reagents needed for PCR, especially oligonucleotides, has been rapidly increasing, and therefore the tracking of the stock in notebooks or spreadsheets and search through frost-encrusted freezer boxes^1^ must be replaced by a far more elegant solution based on computer application.

Oligonucleotides are short, chemically synthesized sequences composed of nucleotide residues, which pair to the complementary nucleic acid section representing its target.^2^ As sequencing becomes more affordable, oligonucleotides are turning into an irreplaceable reagent in a molecular biology laboratory. Oligonucleotides are used for amplifying DNA in different PCR approaches but are also fundamental in many other widespread laboratory techniques, such as molecular cloning, site-specific mutagenesis, and as molecular probes (eg, used as detection probes in fluorescence in situ hybridization, FISH, which enables visualization of the localized gene expression).

Oligonucleotide sequences can be made available from databases such as PrimerBank,^3^ but can also be custom synthesized for specific purposes using designated tools, such as Primer-BLAST, an NCBI Primer designing tool.^4,5^ Properties of oligonucleotides include length, GC content, melting temperature (Tm) range, molecular weight, extinction coefficient, and tendency to form secondary structures that are important for optimizing efficiency, ensuring the proper experimental design and reliable results. Oligonucleotide specificity depends on its length and the sequence of the DNA used in the experiment.^2^ Proper storing and tracking of stored oligonucleotides are important for their stability. For example, unmodified DNA oligonucleotides are stable for 2 years at −20°C, whereas refrigerated at +4°C, they are stable for about a year (>60 weeks) . For cost-effectiveness, oligonucleotides are often shared between laboratory members and, if possible, used for different projects within a larger research group. Therefore, it is important to have a centralized system for oligonucleotide management and tracking.

Nowadays, there are various laboratory reagent management software solutions available, such as LabCollector,^6^ AiO,^7^ OpenFreezer,^8^ MyLabStocks,^9^ LabStoRe,^10^ and LINA.^11^ Even though these solutions are useful for general laboratory use (eg, keeping track of reagents information and stocks management, exporting data), they are not specific for oligonucleotide tracking, ordering, and managing. There are currently no open and/or freely accessible solutions on the market that would comprehensively provide a record of oligonucleotide libraries and fully cover the needs of research and medical laboratories. However, whereas the use of licensed products is necessary and affordable in the industry, it can present a barrier and further financial burden in academic settings.

Here, we present OligoPrime, an open-source and freely accessible web-based software solution, which is devoted to the management of oligonucleotides in small to medium-sized academic laboratories. To ensure security, the software can be hosted on an internal in-house server which can only be accessed from within the internal laboratory network by the laboratory members. Moreover, different access policies and permissions can be defined, which restrict the users of the software to specific roles, such as Student, Researcher, or Administrator. We believe that with this software we can reorganize and simplify working with oligonucleotides in the laboratory and therefore make it faster and more effective, leaving the staff to focus on the content of their work instead of organizational aspects.

## Results

OligoPrime presents software designed to simplify the storage and ordering procedures of oligonucleotides in a laboratory. It is easy to use and has a clear layout and multiple functionalities, which we tried to adapt to several possible laboratory scenarios, such as ordering multiple oligonucleotides from the same supplier in a batch, preventing mixing-up of nucleotides by having an overview over the exact location of storage and their belonging projects and listing the users that recently used a specific oligonucleotide within a project.

The layout of the software is intuitive and simple to use. Moreover, there is a variety of options that allow the user to add and edit oligonucleotide data as well as personalize the user experience. These options are easily accessed to save time on every step of working with oligonucleotides. The software offers different levels of user access; the user can customize the data while all their actions are being recorded in the history section. This functionality also has a protective function of keeping user data safe by following the regulative standard operating procedures (SOPs).

### Functionalities

OligoPrime functionalities are divided into specific parts, which are described in the following section. The full documentation of the software is available at https://oligoprimedocs.readthedocs.io/en/latest/.

#### Login page

All repository users need to log in before viewing or customizing data. New users with assigned roles are created by an administrator. The users are then notified by an e-mail including their username and password. After the first login with the password set by an administrator, the users can change their password.

#### Access to oligonucleotide data library

**Figure 1.**
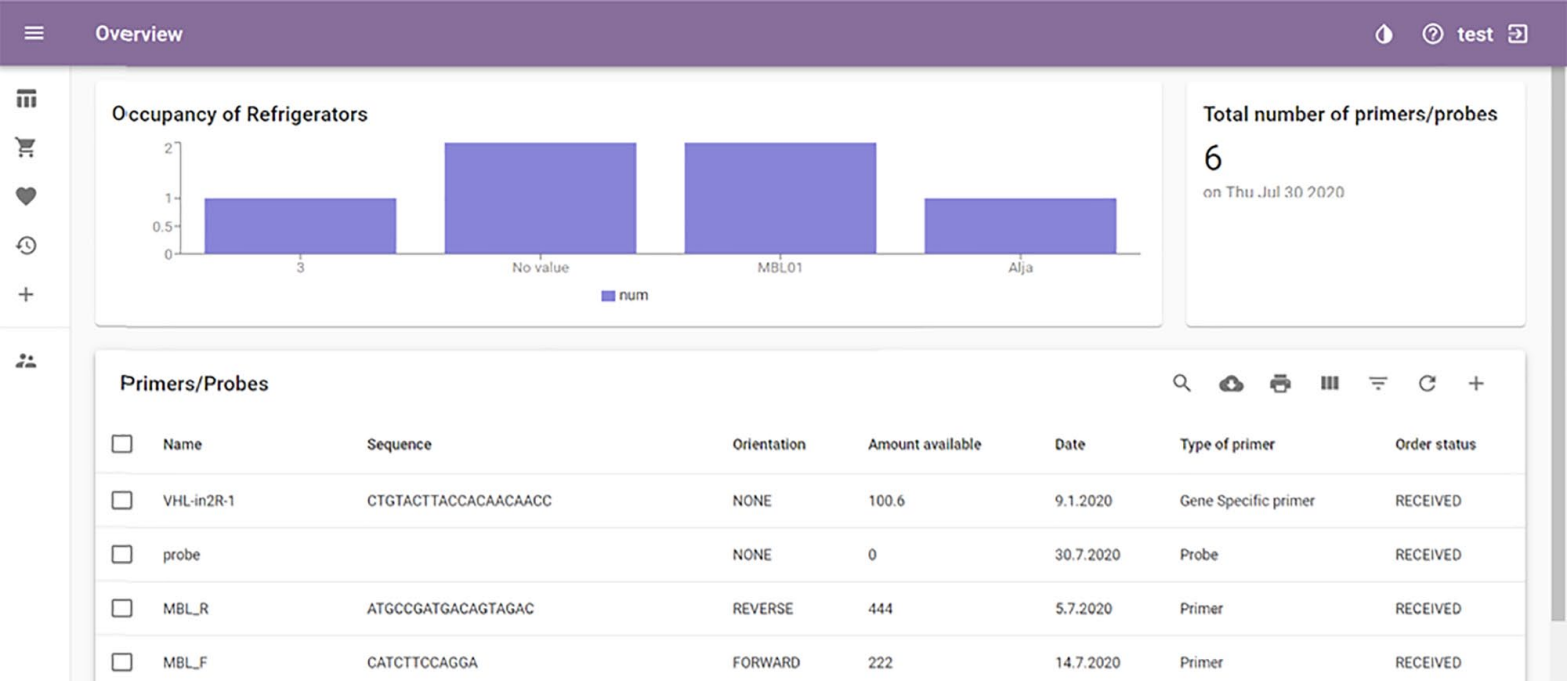
The overview page of the software, showing icons of the left-hand side in descending order: Overview, Orders, Favorites, History, Add primer/probe.

After logging in, the user is presented with the “Overview” page, the main page of the program (See Figure 1).

From the main page, the user can change the theme and access the documentation of the software for further information. The documentation can also be exported and printed in a portable document format (PDF).

The “Overview” page shows general analysis data, including the occupancy of refrigerators and the total number of oligonucleotides. Oligonucleotides are presented in an organized list and are automatically sorted by their generated names in descending order. This order can be modified based on any trait (eg, “Sequence,” “Freezer,” “User.”)

The software allows linking and unlinking of oligonucleotides (eg, forward, reverse, and probe) and their data. The user can add their most frequently used oligonucleotides to a “Favorites” list, which allows faster access to a selection of oligonucleotides. These oligonucleotides appear on the “Favorites” page.

Details of each oligonucleotide can be accessed by clicking on the bar with the oligonucleotide. Oligonucleotides can be modified or deleted by the users with the right level of access. This is described in detail in the “Editing Primer Data” section.

#### Searching and filtering oligonucleotide data

OligoPrime software allows the user to search the database using fast or advanced search options. When using a normal (fast) search, the user can search the database based on all selected attributes simultaneously, depending on the amount selected with the “View columns” option. Advanced search options can be accessed by clicking on the “Filter Table” button and selecting the desired criteria. Filtering can also be combined with searching, which allows us to perform advanced queries in selected subsets.

Filtering is possible using various traits, to mention a few:

- NCBI gene ID
- Generated name of the primer—the name of the oligonucleotide assigned by the software
- Organism—for example, *Homo sapiens, Rattus norvegicus*
- User—the user that added the primer
- Type of primer—for example, TaqMan probe
- Project—the project within which the oligonucleotide was defined
- Supplier—the supplier of the oligonucleotide
- Manufacturer—the manufacturer of the oligonucleotide
- Location (Freezer)—location in the laboratory, for example, Freezer1, Drawer 2, Box 3
- Other properties of a primer, such as melting temperature and dye attached to the primer.

The system also allows the user to search for duplicate oligonucleotides by simply entering the selected sequence into the search field. The system will search for all oligonucleotides with the same sequence, thus list all duplicates. Moreover, the user can also search for oligonucleotides with similar sequences. We can do that by entering a part of a sequence in the search field, from which the system then finds all oligonucleotides that contain this part of the sequence.

#### Sorting oligonucleotide data

Oligonucleotides can be sorted based on certain traits with the “View columns” option and check off the boxes with desired traits, then sorting them in an ascending or descending order. When checking off the boxes, the user can also use the “Select all” option to see all data at the same time.

#### Exporting and printing data

All oligonucleotide data from the database can be exported with the “Download” option. The data is exported in a comma-separated values (CSV) format. The program also allows you to print all data at once.

Specific oligonucleotide data can also be exported by selecting an oligonucleotide, first, selecting “Open data” and then selecting the “Download” option. These data are also exported in a CSV format.

#### Adding new oligonucleotides

New oligonucleotides can be added in 2 ways. The fast and simplified way includes adding oligonucleotides or probes with no obligatory information to fill in (“Add one general”) and the normal way includes all adding options with some obligatory fields (“Add primer”).

The “Add primer” option offers you to add one general oligonucleotide (described in the previous section) or, 1 or 2 (forward and reverse) oligonucleotides at the same time. The software allows you to put in old, already existent oligonucleotide data from the database. There is an option of uploading a file or importing data to the database by selecting the “CSV” option and choosing which document to upload. The software also provides an example of how the data for importing should be structured.

When adding a new oligonucleotide in this way, the user must put in the obligatory data (tagged with an asterisk symbol) before the system allows the user to access the next page. Table 1 represents a summary of obligatory (required) and optional oligonucleotide data.

**Table 1.** Required and optional attributes when adding a new primer or a pair of oligonucleotides.

Required data	Name of the oligonucleotide
	Sequence
	Organism
	Gene
	Position in the reference
	Formulation
	Purification method
	Type of primer
	Application
	5ʹ modification
	3ʹ modification
	Location
	Project
Optional data	NCBI gene ID
	Human genome build
	Length
	Tm (°C)
	Optimal T of annealing (°C)
	GC (%)
	Storing T (°C)
	Probe Sequence (for TaqMan probe)
	Length of amplicon
	Amount available
	Did you check specificity in BLAST?
	Designer
	User
	Supplier
	Manufacturer
	Date
	Comment
	Analysis

When entering the data for a pair of oligonucleotides, common features are put in first, and specific features for each primer second.

When adding a TaqMan probe (by ThermoFisher Scientific^12^), additional parameters specific for this type of oligonucleotide are required, namely, assay ID, size, probe sequence (optional), quencher, and dye. A document, such as the information sheet provided by the manufacturer, can be added as an attachment to the oligonucleotide data.

#### Editing oligonucleotide data

After accessing the oligonucleotide data by opening the oligonucleotide details, data such as the amount available, results of an analysis, and comments can be edited. The user with the assigned role can also edit all other information by clicking on the edit icon and by accessing the “Edit primer” page.

This page allows users to edit the oligonucleotide data or correct any possible mistakes that have been made during the process of adding new oligonucleotides to the repository. This includes basic oligonucleotide properties, oligonucleotide order form, its location in the laboratory, project information, etc.

The “Analysis” field allows the user to link crucial information (eg, graphs or videos) as a part of the analysis to the oligonucleotide data.

Oligonucleotides can be deleted at any time by selecting the “Delete” option. All changes to the data are logged and can be viewed in the “History” section.

#### Ordering oligonucleotides

**Figure 2.**
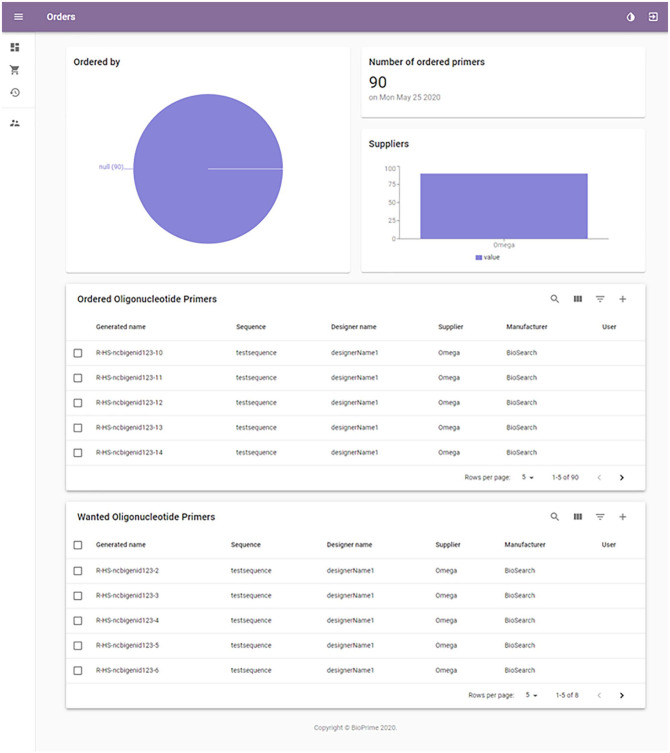
Orders page provides an overview of the recent and pending oligonucleotide orders.

Orders can be made from the “Orders” page (see Figure 2), which can be accessed by clicking on the shopping cart icon. One or 2 oligonucleotides can be ordered simultaneously.

As with the procedure of adding a new oligonucleotide, the required fields should be filled in. When ordering 2 oligonucleotides simultaneously, common features are required first and specific features second for each oligonucleotide. The software allows the user to reorder an oligonucleotide by moving it directly from the library on the “Overview” page to the “Ordered” section of the “Orders” page. This can be done by selecting an oligonucleotide on the “Overview” page and then selecting the “Move to wanted” option. It is advised that if less than 10 µL of an oligonucleotide is available, a new order should be placed immediately. If a mistake has been made while putting in the data, the system sends a warning message.

After completing an order, the date of the order appears in the oligonucleotide details to notify other users searching for that oligonucleotide. Existing orders can be viewed in the “Orders” page, accessible by clicking on the shopping cart icon on the top left part of the page. The “Orders” section also includes the “Wanted” section, where the user can view the oligonucleotides pending to be ordered. This page should be checked so the orders do not overlap. If the oligonucleotide has already been ordered, this is also shown under the “Order status” in oligonucleotide details.

If your order can be ordered in a batch with other oligonucleotides, it can be added as a pending order under the “Wanted” section. This allows other users to add their wishes and when enough material from the same supplier is under “Wanted,” everything can be ordered in a single batch. When an order has been placed, the oligonucleotides from the “Wanted” section are moved to the “Ordered” section.

When an order has arrived, the oligonucleotide can be moved directly to the oligonucleotide repository, but “Project” and “Location of storage” information need to be filled in beforehand. Orders or wanted oligonucleotides can be edited in the same way as the primer data in the library, by clicking on the row with the oligonucleotide and clicking on the “Edit” icon.

#### History view

Users with the appropriate permission (eg, “Student,” “Laboratory Technician,” “Researcher,” and “Administrator”) can access the “History” page and view any recent changes made by other users, including adding or editing oligonucleotide and user data.

#### Adding and customizing users

This function is only visible to an administrator who can add/delete and customize users and their roles. User view is accessed by clicking on the user icon (the bottom option) on the left. The “Admin” page shows the users and their roles with a graphical representation on top of the page showing the distribution of users. When a new user is added, “Full name,” “Username,” “Role,” “Work title,” and “Password” need to be defined by an administrator. The administrator can as well edit existing users and their roles.

### Case study

We describe a potential scenario, in which the user wants to add a pair of oligonucleotides with the parameters as described in Tables 2, 3, and 4. The reader can follow the same process as described here using a prototype implementation of OligoPrime available at http://oligoprime.xyz/ using the login credentials—*username*: admin, *password*: admin.

**Table 2.** Common parameters for a pair of hypothetical oligonucleotides.

Organism	*Homo sapiens*
Gene	catX
NCBI gene ID	Ctx
Human genome build	NCBI Build 36.1
Length of amplicon	500
Type of primer	TaqMan probe
Did you check specificity in BLAST?	Yes
Probe sequence	ATGCCGATGACAGTAGAC
Size	M
3ʹ Quencher	QSY
5ʹ Dye	VIC
Formulation	Resuspended in 1x TE
Storing T	−20
Purification method	HPLC
Pack type	1 tube
Amount available	300 µl
Concentration	10 µM
Project	Project X
Application	RT-qPCR-TaqMan
Application comment	/
Designer name and surname	John Doe
Publication	/
Link to database	/
Supplier	OligoSupply
Manufacturer	OligoProduce

**Table 3.** Parameters for the forward sequence.

Name of primer	CatX_forward_mutA
Sequence	ACTAGCCGATACAGATCGATCAGAT
Tm	65
GC %	45
Optimal T of annealing	66
Position in the reference	No data
5ʹ modification	None
3ʹ modification	None
Location	Fridge: Jane, Drawer: 3, Box: New primers
Additional information	/

**Table 4.** Parameters for the reverse sequence.

Name of primer	CatX_reverse_mutA
Sequence	ACGTGCGACGATCGAGCGATCGAGCTA
Tm	65
GC %	49
Optimal T of annealing	66
Position in the reference	No data
5ʹ modification	None
3ʹ modification	None
Location	Fridge: Jane, Drawer: 3, Box: New primers
Additional information	/

**Figure 3.**
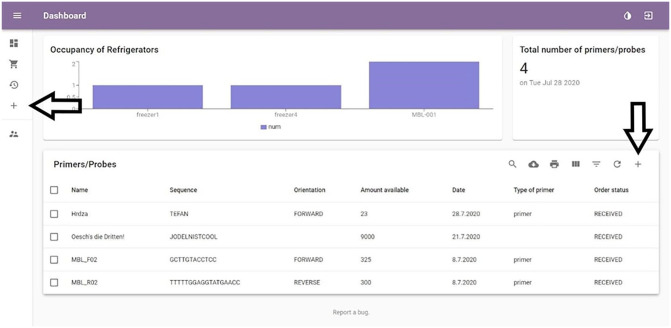
Overview page. The left arrow is pointing to the “Add one general” button and the right arrow is pointing to the “Add primer” button.

Firstly, the user needs to log in using a valid username and password. In our case, we will use the username “admin” and password “admin.” We are presented with the “Overview” page with statistical data on top and a list of oligonucleotides on the bottom. We could do a simple add by clicking on the “Add one general” icon on the left, but instead, we will add the data using the “Add primer” option on the top right, which will not allow us to proceed without adding the obligatory data. The screenshot of both options is represented in Figure 3.

**Figure 4.**
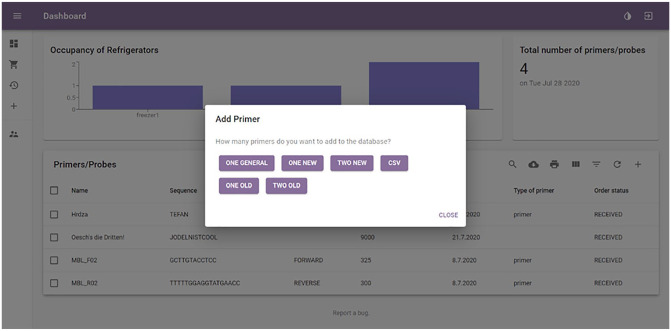
Pop-up window, offering different options to add new or existing oligonucleotides. “One general” option allows data insertion without obligatory attributes.

A new window opens, asking if we want to add 1 or 2 new oligonucleotides or if we want to just add an old one (see Figure 4). We choose “two new.”

**Figure 5.**
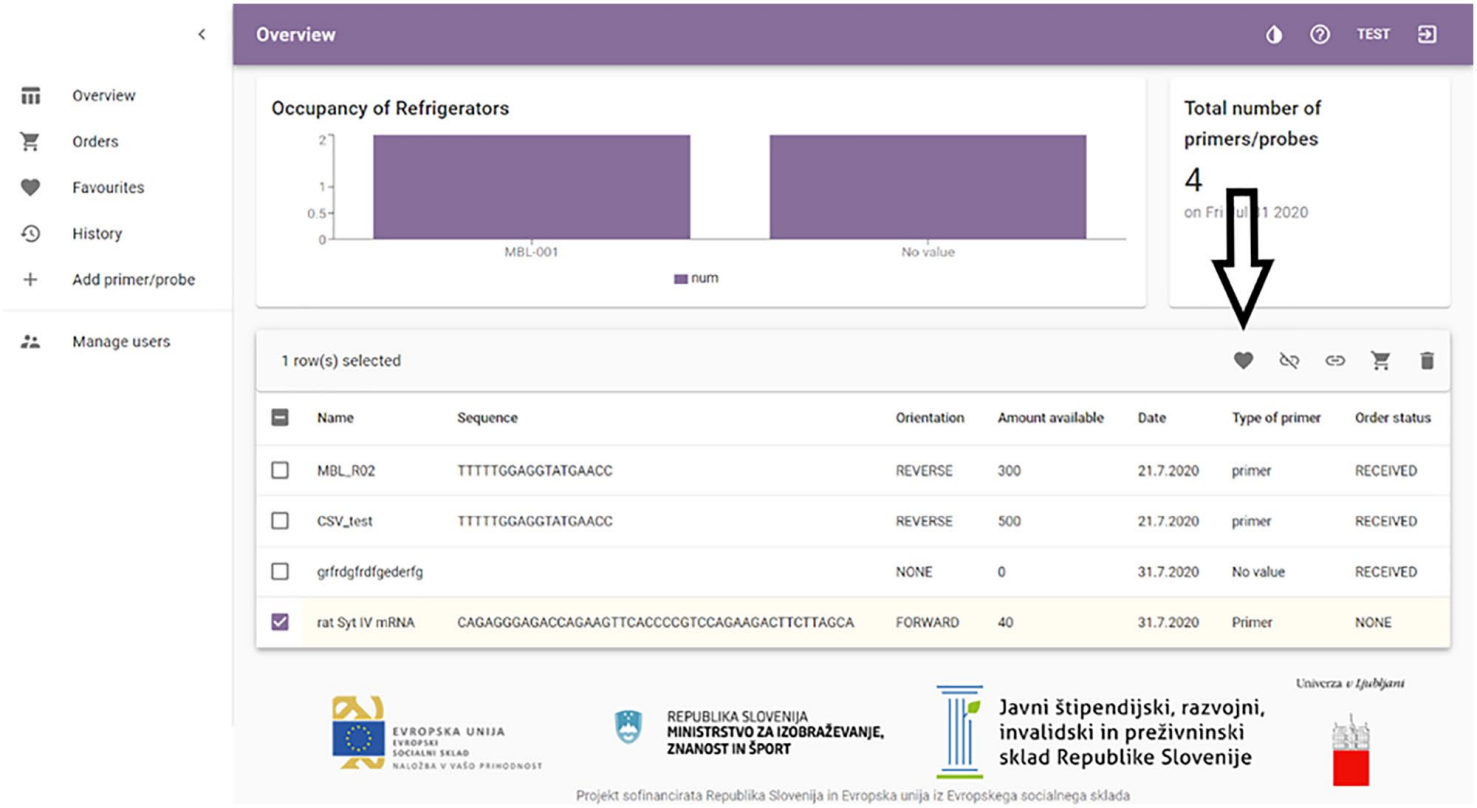
Overview page with the arrow pointing to “Add oligonucleotides to favorites” icon.

In the next window, we insert the common features in the top section. In our case it includes “Gene,” “NCBI gene ID,” “Human genome build,” “Length of amplicon,” “Probe sequence,” “Size, 3’ Quencher,” “5’ Dye,” “Formulation,” “Storing Temperature,” “Purification method,” “Pack type,” “Amount available,” “Concentration,” “Project,” “Application,” “Designer name and surname,” “Supplier,” and “Manufacturer.” The contents of these fields can be viewed at the beginning of this section. In the lower part of the page, we will put in data specific for each individual oligonucleotide. In our case, this includes “Name of primer,” “Sequence,” “Tm,” “GC %,” “Optimal T of annealing” and the location of each oligonucleotide. When we submit the data, the oligonucleotide appears on the “Overview” page. The oligonucleotides are now linked because their data was put in simultaneously. We can add a newly added oligonucleotide from the “Overview” page to “Favorites” by selecting the primer and clicking the “Favorites” icon on the right (Figure 5).

## Methods

OligoPrime implementation is based on three-tier client-server architecture. With this approach, we have achieved many benefits both in development and production environments by modularizing the user interface, business logic, and data storage layers. Thus, simultaneous developments of separate segments were possible, but the main benefit is that each tier can be deployed on different heterogeneous and distributed platforms, which is essential for load balancing, reliability, and availability.

### Software tools and packages

As mentioned above, we divided the application into 3 layers (presentation tier, application tier, and data tier). On each layer we used technologies and frameworks suitable for them. Because OligoPrime is a web application, we were choosing between JavaScript libraries for the presentation layer and decided to go with React.js^13^ which is an open-source JavaScript library for building user interfaces (UI). On the application layer we decided to use Java, which is a robust programming language, alongside with Java framework Dropwizard,^14^ used for developing a backend for web applications. For data storage we used a popular and arguably most advanced open-source relational database PostgreSQL.^15^

### Back end implementation

Core architectural style used in our backend application is representational state transfer (REST), which is providing resources addressable through uniform resource locator (URL) addresses, using HTTP (Hyper Text Transfer Protocol) to transfer files. We developed a REST application programming interface (REST API), a web service to which the frontend of the application is connected to retrieve and store data from/into the database. For implementation of the API we used Dropwizard. Dropwizard pulls together necessary, stable and reliable libraries from the Java ecosystem that allows faster and more robust development. Dropwizard also includes a jDBI (Java Database Interface) module for convenient and idiomatic interaction with relational databases *via* SQL (Structured Query Language). We applied jDBI to connect our backend application to the database.

### Front end implementation

The key framework used in our frontend application is React.js, JavaScript library for building user interfaces. Because we wanted the web application to feel like a native application, we created a single-page application (SPA) using the React Router^16^ library, which is already integrated into React.js and is ready to use straight out of the box. The biggest benefit, in contrast to traditional websites, is Client-side rendering, which handles the routing dynamically without refreshing the page every time a user requests a different route. Meaning that navigating an SPA does not involve going to an entirely new page, but instead the whole application lives inside the browser and interfaces with our backend application *via* RESTful API to retrieve or send data. While building user interfaces, it is essential to also consider user experience (UX). For our web application, we used React.js library Material Design^17^ for fast development and uniform design. Material Design consists of components which are user friendly and intuitive, and we integrated them into our application.

### Requirements

Recommended requirements for the server on which the backend application will be running are listed below.

1. Operating system: Ubuntu 20.4 LTS
2. RAM: 4 GB (or more)
3. CPU: 4 core (or more)
4. Storage: 100 GB (or more)

Recommended client requirements:

1. CPU: 4 core (or more)
2. RAM: 4 GB (or more)
3. Browser: Google Chrome (Recommended), Mozilla Firefox, Safari

### Installation

As previously mentioned, we used the three-tier architecture with each layer having its own application. For each application (backend and frontend), we created docker images and pushed them to Docker Hub.^18^ The installation needs to be performed by an administrator on the server where the latest version of Docker^19^ and Docker Compose^20^ are installed. We also prepared a *docker-compose.yml* file that pulls the images from Docker Hub and starts hosting the applications. A more detailed installation manual together with all the files required for the installation are available at https://github.com/OligoPrime/documentation/blob/master/docs/installation.md. When the application is successfully deployed on the server, a user can access it *via* a web browser by entering the server’s hostname into the browser’s address bar.

## Discussion and Conclusions

Previously, laboratory reagents could be successfully managed using laboratory records on paper or using one of the general-purpose spreadsheet tools. However, with the growing number of techniques and necessary reagents, this is no longer the case. It is now clear that implementing automated systems of electronic records will simplify experimental work and will ensure the successful running of research laboratories. An example of such a tool is OligoPrime, a software dedicated for specific use: management and tracking of oligonucleotides. OligoPrime is free, straightforward, and easy-to-use, and is intended for use in small to medium-sized laboratories. It supports a transparent view of the stored oligonucleotides, their quantities, date of order/purchase, and history of their use. Furthermore, the software can be helpful when using both PCR and non-PCR techniques, such as site-specific mutagenesis or in situ hybridization, since the oligonucleotide data include information specific for most wide-spread techniques used in a laboratory setting. What distinguishes OligoPrime from the other 2 freely available solutions LabStoRe and OpenFreezer, is that while they are broadly oriented to laboratory reagents, in general, OligoPrime is dedicated to oligonucleotide management only. Moreover, even though the proposed software can be used directly out-of-the-box, its code is freely available, which means that its potential users can adapt its functionality according to their own needs.

Presently, OligoPrime has already been successfully applied in the laboratories of the Medical Centre for Molecular Biology, Institute of Biochemistry and Molecular Genetics at the Faculty of Medicine, University of Ljubljana. However, due to its simplicity of use and affordability, we believe that OligoPrime will be of interest to researchers of different fields of biological and biomedical research. The OligoPrime software will provide a foundation for automatization of oligonucleotide management and tracking with minimal human input.
